# Added predictive value of prehospital measurement of point-of-care lactate in an adult general EMS population in Sweden: a multi-centre observational study

**DOI:** 10.1186/s13049-024-01245-7

**Published:** 2024-08-20

**Authors:** Carl Magnusson, Johan Herlitz, Christer Axelsson, Robert Höglind, Elin Lökholm, Thea Hillberg Hörnfeldt, Agnes Olander, Joakim Björås, Magnus Andersson Hagiwara, Pär Wennberg

**Affiliations:** 1 Department of Prehospital Emergency Care, Sahlgrenska University Hospital, Gothenburg, Sweden; 2 Centre for Prehospital Research, Faculty of Caring Science, Work Life and Social Welfare, University of Borås, Borås, Sweden; 3 Department of Molecular and Clinical Medicine, Institute of Medicine, Sahlgrenska Academy, University of Gothenburg, Gothenburg, Sweden; 4 Emergency Medical Services, Skaraborg Hospital, Skövde, Sweden; 5 Department of Health and Society, Kristianstad University, Kristianstad, Sweden; 6 Department of Research, Development, Education and Innovation, Sahlgrenska University Hospital, Gothenburg, Sweden; 7 School of Health Sciences, Jönköping University, Jönköping, Sweden

**Keywords:** Prehospital, Point-of-care, Patient assessment, Emergency medical service, Lactate

## Abstract

**Background:**

Emergency medical services (EMS) personnel must rapidly assess and transport patients with time-sensitive conditions to optimise patient outcomes. Serum lactate, a valuable in-hospital biomarker, has become more accessible in EMS settings through point-of-care (POC) testing. Although POC lactate levels are valuable in specific patient groups, its broader application in EMS remains unclear. This study assessed the additional predictive value of POC lactate levels in a general adult EMS population.

**Methods:**

This prospective observational study (March 2018 to September 2019) involved two EMS organisations in Västra Götaland, Sweden. Patients were triaged using the Rapid Triage and Treatment System (RETTS). POC lactate levels were measured using StatStrip Xpress devices. Non-consecutive patients who received EMS and were aged 18 years and above were available for inclusion if triaged into RETTS levels: red, orange, yellow, or green if respiratory rate of ≥ 22 breaths/min. Outcomes were adverse outcomes, including a time-sensitive diagnosis, sequential organ failure assessment (SOFA) score ≥ 2, and 30-day mortality. Statistical analyses included descriptive statistics, imputation, and regression models to assess the impact of the addition of POC lactate levels to a base model (comprising patient age, sex, presence of past medical conditions, vital signs, pain, EMS response time, assessed triage condition, and triage level) and a RETTS triage model.

**Results:**

Of 4,546 patients (median age 75 [57, 84] years; 49% male), 32.4% had time-sensitive conditions, 12.5% met the SOFA criteria, and 7.4% experienced 30-day mortality. The median POC lactate level was 1.7 (1.2, 2.5) mmol/L. Patients with time-sensitive conditions had higher lactate levels (1.9 mmol/L) than those with non-time-sensitive conditions (1.6 mmol/L). The probability of a time-sensitive condition increased with increasing lactate level. The addition of POC lactate marginally enhanced the predictive models, with a 1.5% and 4% increase for the base and RETTS triage models, respectively. POC lactate level as a sole predictor showed chance-only level predictive performance.

**Conclusions:**

Prehospital POC lactate assessment provided limited additional predictive value in a general adult EMS population. However, it may be beneficial in specific patient subgroups, emphasizing the need for its judicious use in prehospital settings.

**Supplementary Information:**

The online version contains supplementary material available at 10.1186/s13049-024-01245-7.

## Background

When there is high suspicion of a time-sensitive condition, one of the primary objectives of emergency medical services (EMS) is to assess a patient at the scene and transport them to the hospital as quickly as possible to reduce any delay to receiving definitive care. Prehospital EMS presents unique challenges because delayed and misinformed decisions can significantly impact patient outcomes. However, the absence of real-time diagnostic tools often limits the ability of EMS staff to identify conditions, such as sepsis, shock, and organ dysfunction, at the earliest stage. These conditions are characterised by a rapid deterioration in health, making early detection crucial for effective interventions.

At the hospital level, serum lactate is routinely used as a biomarker for additional information in the diagnostic workup of critically ill patients, including those with sepsis, among other time-sensitive conditions [1–6]. With the introduction of point-of-care (POC) tests, the availability of lactate measurements in the EMS setting has increased. However, the current standard of prehospital care still relies primarily on clinical assessment and basic vital signs (VS), often leaving underlying metabolic disturbances undetected until hospital arrival. Although some POC devices for lactate measurement exist, they are not yet widely adopted in prehospital settings because of concerns regarding accuracy, ease of use, and practicality. However, several studies have reported the usefulness of POC lactate assessment in the EMS setting for specific patient groups in the early phase of trauma, indicating the need for blood products, intensive care, hospital admission and accuracy in trauma level activation [7–10]. Furthermore, metabolic acidosis can predict short-term mortality in out-of-hospital cardiac arrests, and reduced lactate clearance can predict short-term mortality in sepsis and septic shock [11–16]. In non-critical patients, POC lactate has a potential predictive value after tonic–clonic seizures [17]. However, neutral results or similar informative value to that of routine clinical assessment have been reported in patients with sepsis [18, 19].

Consequently, there is limited evidence regarding the value of POC lactate levels in the general EMS patient population. Therefore, the overall aim of this study was to evaluate the added value of POC lactate to the patient assessment and triage in adult patients in the general EMS population.

## Methods

### Study design

This study was conducted as a multi-centre investigation encompassing patients who initiated contact with Swedish EMS and for whom an ambulance was subsequently dispatched. The study period was March 2018 to September 2019.

### Study setting

Two EMS organisations within the Västra Götaland region of Sweden (the Department of Prehospital Emergency Care at Sahlgrenska University Hospital in Gothenburg, and the Department of Prehospital Emergency Care in Skaraborg) participated in the study. These EMS organisations cover both urban and rural areas. The EMS organisations are tax-funded under the authority of the region, serve a community of approximately 960,000 inhabitants, and respond to over 118,000 assignments annually. Of these, approximately 83,000 were considered primary assignments involving patient assessments at the scene.

### Patient assessment

At the dispatch centre, an ambulance is dispatched with one of three priorities: Priority 1 (life-threatening), Priority 2 (urgent), and Priority 3 (transport). The ambulance is crewed by at least one registered nurse (RN) according to national legislation, and the majority of the RNs in the study organisations have an additional year of post-graduate education, specialising in prehospital emergency care, anaesthesia care, or intensive care. At the scene, patients are assessed following national and regional guidelines and recommendations, including Advanced Medical Life-Support (AMLS) and Prehospital Trauma Life-Support [20, 21]. Based on the clinical assessment, the RN triage patients in the prehospital setting for a seamless transition between the prehospital setting and the emergency department (ED). The most common triage system in Sweden is Rapid Triage and Treatment System (RETTS), which was initially developed at the Sahlgrenska University Hospital ED. RETTS is licensed and maintained by Predicare AB, a company that develops decision-support systems. RETTS is a five-level triage system that includes the most common ED presentations. The patient’s level of severity is determined by emergency signs and symptoms (ESS) and VS. For example, the ESS contains risk factors and/or electrocardiogram findings which may induce a higher triage level. The highest triage level of ESS or VS instigates the final triage level in which order the patient should be managed in the ED. The EMS organisations participating in the study utilises the following RETTS triage levels classified from highest to lowest severity: ‘**Red'** (life-threatening, immediate intervention needed), **'Orange'** (very urgent, potentially life-threatening), **'Yellow'** (urgent, not life-threatening), **'Green'** (non-urgent, not life-threatening). Additionally, a level below green is used in the ED but not in the EMS: **'Blue'** (non-acute, minor). Patients triaged as **'Yellow'**, **'Green'**, and **'Blue'** can wait for evaluation by an ED physician without significant risk of deterioration.

### Prehospital POC lactate measurements

POC lactate was measured and registered in electronic patient medical records (Ambulink). Lactate in whole blood was obtained from a capillary sample at the scene or en route using a Stat Strip Xpress (SSX) (Nova Biomedical, Waltham, MA, USA). The SSX device measurement interval is 0.3 mmol/L to 20.0 mmol/L (3–180 mg/L). The amount of blood required is 0.6 µL and the analysing period is 13 s. At the time of the study, the SSX was determined to be feasible for use in the prehospital setting and at a low cost per test. Reproducibility and concordance with standard laboratory devices have been demonstrated in previous studies with acceptable results [22, 23]. However, a proportional negative bias has been reported at higher concentrations [24, 25]. The SSX was validated by the EMS before the initiation of the study, with the support of the Central Laboratory at Sahlgrenska University Hospital.

### Study population

Patient inclusion was non-consecutive and based on the patient assessment undertaken by the EMS RN. Prehospital POC lactate was introduced in the EMS organisations as part of standard care before the start of the study. Educational efforts were undertaken both in the form of physical meetings with all EMS crews and written instructions of test procedures and inclusion criteria. During the study period, reminders were sent at frequent intervals. The EMS RNs were also instructed to obtain a second POC lactate level if the first measurement was higher than 2.0 mml/L. The inclusion criteria were: (1) patients assessed by the EMS RN who were 18 years and above and (2) triaged to a RETTS triage level of red, orange, yellow or green with a measured respiratory rate of 22 breaths per minute or above according to the sequential organ failure assessment (SOFA) score [26]. The purpose of inclusion across all triage levels was to potentially identify a broad population for which POC lactate could add information to patient assessment. The size of the study population was determined based on approximately 5,000 available POC tests. During the study period, 5,259 patients underwent POC lactate measurements. Among them, 713 patients were excluded for the following reasons: age < 18 years (n = 71), erroneous measurement (n = 25), no social security number (n = 48), cardiac arrest (n = 11), missing triage level (n = 72), green triage level with a respiratory rate < 22 breaths/min (n = 362) and lost to follow-up (n = 124).

### Outcomes

We assessed the following adverse outcomes:

1. A diagnosis of a time-sensitive condition according to the international classification of disease codes (ICD-10-SE) related to prehospital patient presentation. A time-sensitive diagnosis was determined based on definitions in previous work [27]. Furthermore, all diagnoses in the study population were reviewed independently by four of the authors. Diverging classified diagnoses were discussed until a consensus was reached with support from specialist physicians.
2. Patients with infection determined by the ED physician and a SOFA score of ≥ 2, thus meeting the Sepsis-3 criteria. The baseline SOFA score was assumed to be zero unless the medical records indicated pre-existing organ dysfunction. In such cases, an acute change of two or more points from the baseline SOFA score was used to determine if the criteria were met [26]. This part included a review of in-hospital patient notes for up to 48 h by one reviewer per site. Interreliability analysis was performed on 100 patients with an acceptable Cohen’s kappa coefficient (0.857). The Strama national application was used for SOFA calculations [28].
3. Short-term mortality, defined as death within 30 days by any cause. This was calculated from the date of the prehospital patient encounter.

### Statistical analysis

Descriptive statistics were reported as frequencies, percentages, medians, and quartiles (Q1 and Q3). We included variables associated with the outcome in the prehospital setting and developed a base model that included patients age and sex, presence of past medical conditions, VS, perceived level of pain, assessed triage condition, triage level and EMS response time. We also included study sites defined as urban or rural as internal validation for distance to the hospital and prior knowledge of the different utilisation of prehospital resources between more densely populated urban areas. To avoid excess ties in the numeric pain scale (NRS) (0–10 measuring pain), the scale was refactored from a continuous scale to an ordinal scale representing pain levels 0–10.

Five models were fit. These were: (1) base model, (2) base model + POC lactate, (3) triage level (RETTS), (4) triage level + POC lactate; and (5) POC lactate as the only predictor. The likelihood ratio test was used to determine whether POC lactate in the prehospital setting added any value in terms of the prediction of a time-sensitive condition compared to the base model. Missing data were infrequent (3–5%) for most of the variables except for NRS which had 45% missing which were assumed to be missing at random. Missing data were imputed using predictive mean matching with a chained equation approach including all variables as candidate predictors for imputation. Fifty datasets were imputed and analysed separately. Nonlinear relationships were modelled using restricted cubic splines with knots. The number of knots was determined using the Akaike information criterion resulting in the use of four knots. We added POC lactate to the base regression model and compared these two models, as well as a reduced model with triage levels only. The models were validated and calibrated by bootstrapping (Additional file 1). The corrected validated indices for the averaged likelihood ratio, Nagelkerke’s pseudo R^2^, and the Somer’s Dxy. This was reported together with the c-index (concordance probability) which is equivalent to Somers´ Dxy rank correlation between predicted and observed and with the area under the receiver operating characteristic (ROC) curve (Dxy = 2(c-index − 0.5)).

The added predictive value of POC lactate level was reported as a fraction of new information based on the ratio of explainable variance before and after POC lactate was added to the model. Receiver operating characteristic (ROC) curves were plotted and a nomogram of the fitted base model, including POC lactate levels, was depicted (Additional file 2). All analyses were performed using R Statistical Software (v4.3.1; R Core Team 2023) and the packages Regression Modelling Strategies (RMS) (v6.7-1) [29] and ggstatsplot (v0.12.3) [30].

## Results

**Fig. 1:**
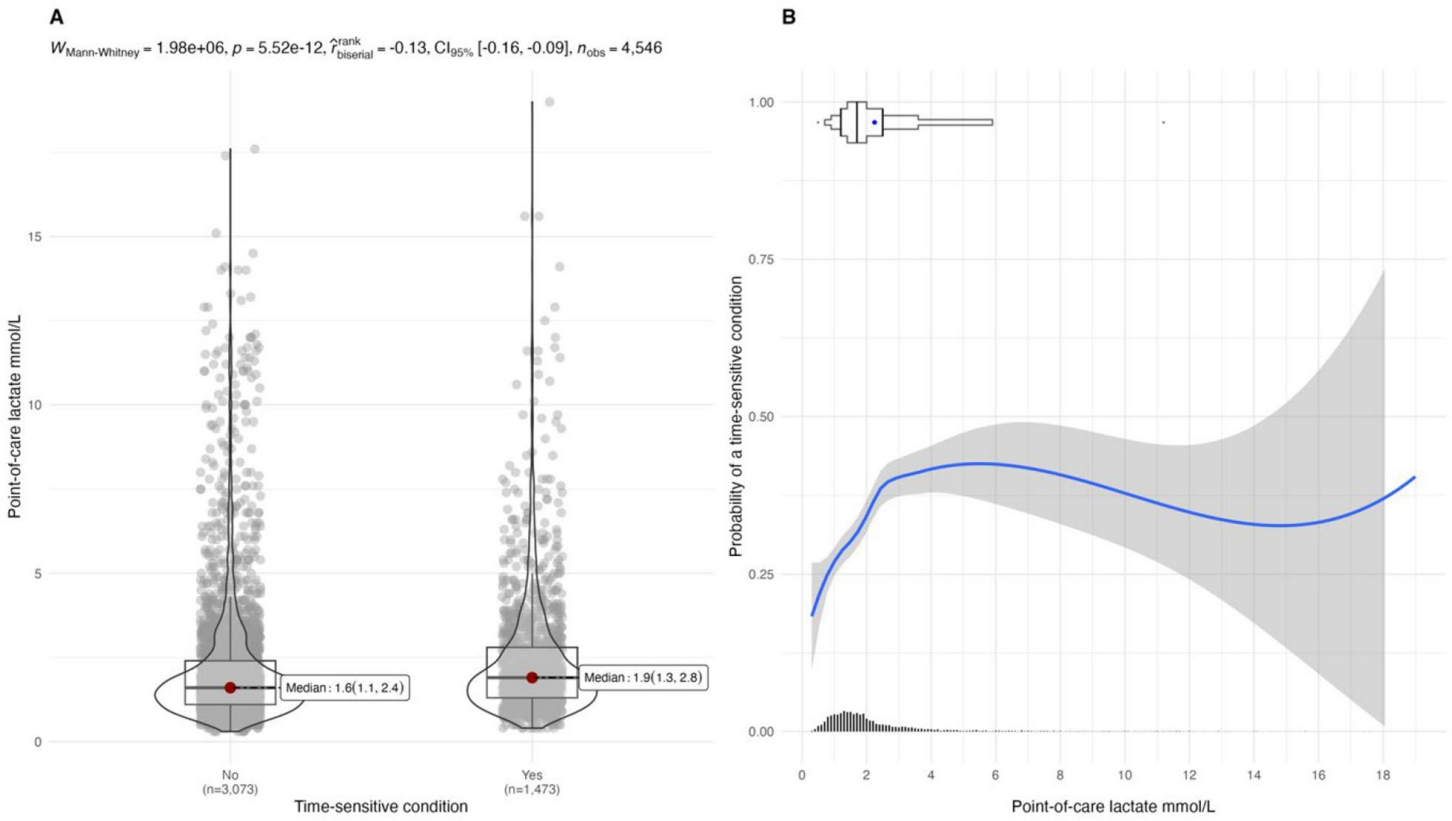
**A**: distribution, median (Q2) and quartiles (Q1, Q3) of point-of-care lactate in non-time-sensitive and time-sensitive conditions. **B**: probability of a time-sensitive condition with 95% confidence intervals for continuous point-of-care lactate mmol/L

**Fig. 2:**
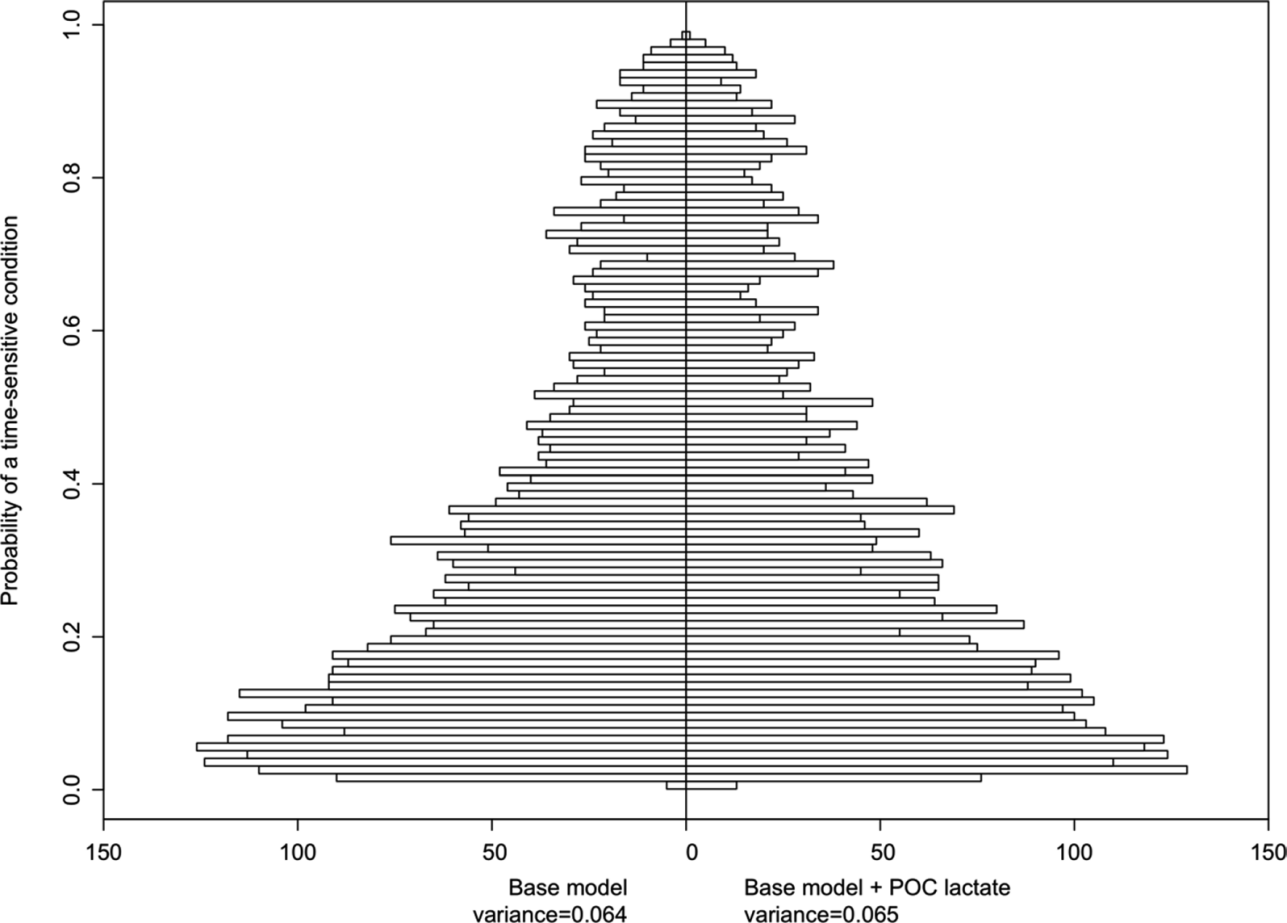
Back-to-back histogram compares the distribution (n = 4,546 on each side) of predicted probabilities from two models: the base model (left) and the base model with POC lactate (right). If POC lactate adds important information the histogram widens. If the histograms to the left and and to the right have similar distributions (i.e., similar shapes and variances), it suggests that POC lactate might not have substantially changed the predicted probabilities. It implies that the other variables in the base model may already capture most of the variability in the outcome

A total of 4,546 patients were included of which 1,473 (32.4%) had time-sensitive conditions. Among them, 20.4% had a time-sensitive diagnosis, 12.5% fulfilled the SOFA criteria, and 7.4% died within 30 days. The median age of the patients was 75 (57, 84) years, and 49% were males. Among all patients, the median POC lactate was 1.7 (1.2, 2.5) mmol/L (Table 1). There was a difference in median POC lactate between the non-time-sensitive and time-sensitive groups (1.6 [1.1, 2.4] mmol/L and 1.9 [1.3, 2.8] mmol/L, respectively) (Fig. 1A). The probability of a time-sensitive condition increased with increased lactate levels but showed a non-monotonic distribution with decreasing probability at higher POC lactate levels (Fig. 1B). We assessed the inter-quartile odds ratio (IQOR) between the POC lactate and a time-sensitive condition for patients in the 50th percentile of the lower quartile (25th percentile) versus those in the 50th percentile of the upper quartile (75th percentile) of the POC lactate distribution. The inter-quartile range was between 1.2 and 2.5 mmol/L. The odds ratio associated with this range was 1.26 (95% CI [1.09,1.46]), indicating that a one-unit increase in POC lactate within this range is associated with a 26% increase in the odds of a time-sensitive condition. In the visual presentation of the probability of a time-sensitive condition for the base model with the addition of lactate to the model, discernible alterations in the distribution were not observed when adding POC lactate (Fig. 2).

**Table 1:** Patient age, sex, medical history, EMS location, EMS response time, triage level, assessed condition, vital signs, perceived pain and lactate in relation to time-sensitive condition

	N	Non time-sensitive	Time-sensitive condition	Total
		N = 3073	N = 1473	N = 4546
Age in years	4546	72 (51,82)	79 (69,86)	75 (57,84)
Sex				
Male	4546	1400 (45.6)	811 (55.1)	2211 (48.6)
Medical history	4546			
Malignancy		585 (19.0)	406 (27.6)	991 (21.8)
Diabetes		506 (16.5)	370 (25.1)	876 (19.3)
Liver disease		133 (4.3)	70 (4.8)	203 (4.5)
Kidney disease		447 (14.5)	322 (21.8)	769 (16.9)
Geographical area				
Urban		2066 (67.2)	830 (56.3)	2896 (63.7)
Prehospital delay minutes	4546	21.4 (13.4,34.6)	19.1 (12.8,30.9)	20.6(13.2,33.6)
Triage level	4546			
Green		24 (0.78)	5 (0.34)	29 (0.64)
Yellow		1343 (43.7)	334 (22.7)	1677 (36.9)
Orange		1432 (46.6)	635 (43.1)	2067 (45.5)
Red		274 (8.9)	499 (33.9)	773 (17.0)
Assessed condition	4546			
Abdominal pain		441 (14.4)	147 (10.0)	588 (12.9)
Arrhytmia		82 (2.7)	32 (2.2)	114 (2.5)
Backpain		48 (1.6)	5 (0.3)	53 (1.2)
Chestpain		349 (11.4)	97 (6.6)	446 (9.8)
Dizziness		187 (6.1)	20 (1.4)	207 (4.6)
Dyspnoea		317 (10.3)	246 (16.7)	563 (12.4)
Endocrine/diabetes		48 (1.6)	30 (2.0)	78 (1.7)
Extremity pain		58 (1.9)	14 (1.0)	72 (1.6)
Fever/infection		339 (11.0)	418 (28.4)	757 (16.7)
GI-bleeding		34 (1.1)	28 (1.9)	62 (1.4)
Headache		38 (1.2)	7 (0.5)	45 (1.0)
Intoxication		40 (1.3)	41 (2.8)	81 (1.8)
Psychiatric dis		18 (0.6)	4 (0.3)	22 (0.5)
Seizures		223 (7.3)	22 (1.5)	245 (5.4)
Stroke/TIA		103 (3.4)	139 (9.4)	242 (5.3)
Transient loss of unconsoiusness		143 (4.7)	26 (1.8)	169 (3.7)
Trauma		308 (10.0)	59 (4.0)	367 (8.1)
Unspecific		233 (7.6)	123 (8.4)	356 (7.8)
Urinary/Gyn		64 (2.1)	15 (1.0)	79 (1.7)
Vital signs				
Respiratory rate	4502	20 (16,22)	22 (18,28)	20 (16,24)
Oxygen saturation	4542	97 (95,98)	94 (90,97)	96 (93,98)
Heart rate	4541	86 (75,102)	95 (80,110)	89 (75,105)
Systolic blood pressure	4513	140 (120,155)	134 (110,155)	139 (120,155)
Body temperature	4420	37.0 (36.6,37.5)	37.3 (36.7,38.4)	37.1(36.6,37.7)
Altered consiousness	4540	167 (5.4)	252 (17.1)	419 (9.2)
Pain				
Numeric rating scale	1691	0 (0,6)	0 (0,4)	0 (0,5)
Lactate mmol/L	4546	1.6 (1.1,2.4)	1.9 (1.3,2.8)	1.7 (1.2,2.5)

The lower quartile (Q1), the median (Q2), and the upper quartile (Q3) for continuous variables

Numbers and percentages for categorical variables

N is the number of non-missing values

Prehospital delay in minutes: EMS response time from dispatch received call to EMS arrival at the scene

EMS: Emergency medical service; TIA: Transient icheamic attack; mmol/L: millimol per litre

A likelihood ratio (LR) test was performed between base model and base model + POC Lactate, yielding a p-value of < 0.001, suggesting that a difference between the models was present and that POC lactate does add value to the performance of the model. For Nagelkerke’s pseudo R^2^, there was almost no difference between base model and base model + POC Lactate in terms of absolute difference (0.349 and 0.352, respectively) and Brier score (0.16 and 0.159, respectively) (Table 2).

**Table 2:** Corrected indices of model difference and performance

	Base model	Base model + POC lactate	RETTS	RETTS + POC lactate	POC lactate
Somer’s Dxy	0.621	0.623	0.334	0.368	0.143
Nagelkerke’s R2	0.349	0.352	0.136	0.144	0.018
Brier score	0.16	0.159	0.196	0.194	0.216
Gini's mean difference	1.589	1.597	0.669	0.754	0.282
Overall quality	0.287	0.289	0.102	0.109	0.012

Overall quality: Logarithmic accuracy score, a scaled version of the log-likelihood achieved by the predictive model

POC: Point-of-care; RETTS: Rapid emergency triage and treatment system

We assessed the contribution of lactate to the base model by calculating the total fraction of new information, defined as (1 − relative explained variation). We found that this fraction was 1.5%. The relative explained variation was determined by the ratio of the variance of the base model to the variance of the base model + POC lactate. This calculation yielded a relative explained variation of 0.9846 (0.064/0.065).

**Fig. 3:**
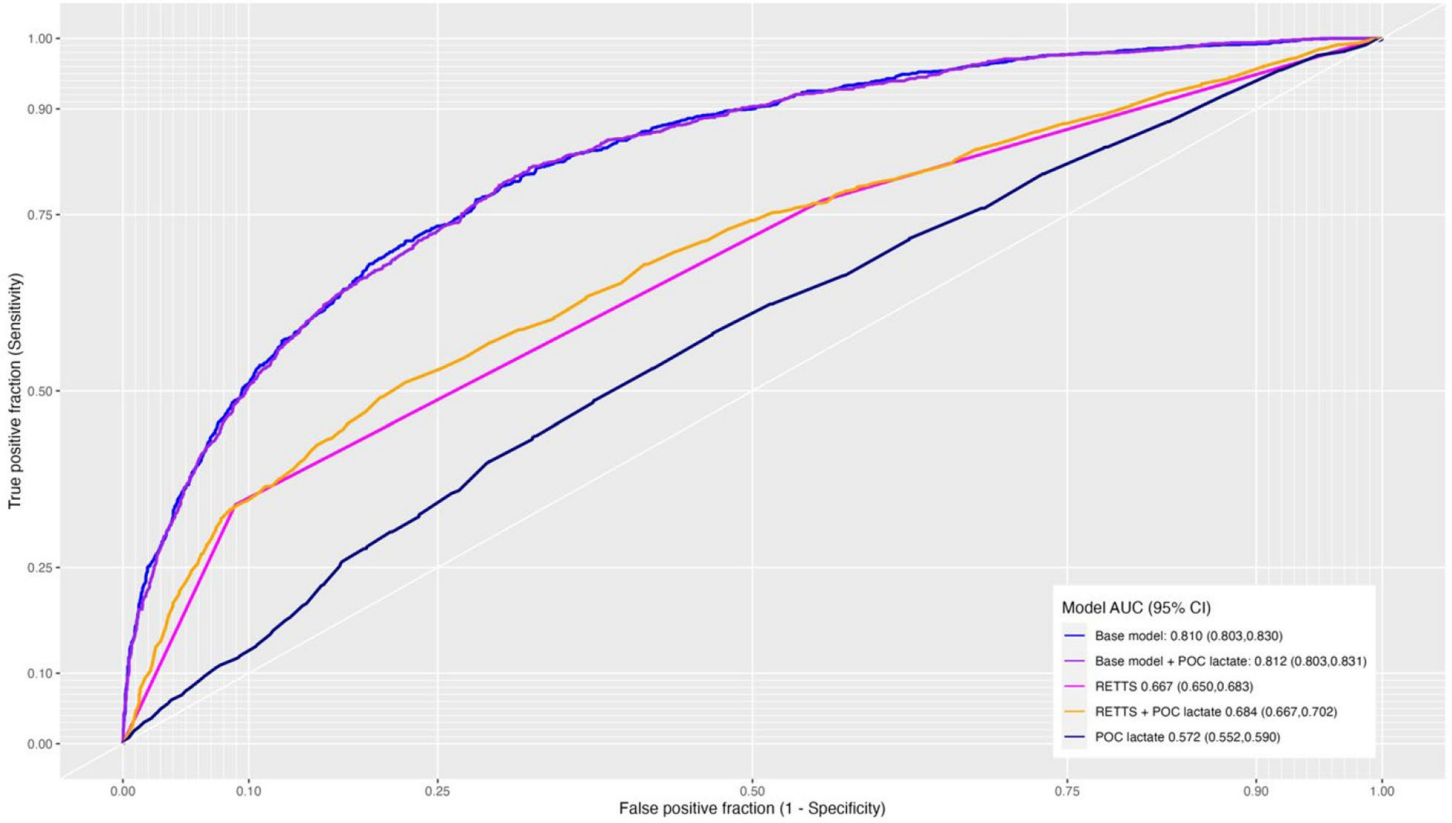
Receiver Operating Characteristic (ROC) curve illustrating the performance of the five models in classifying individuals with and without a time-sensitive condition. The curve shows the trade-off between the true positive rate (sensitivity) and the false positive rate (1—specificity) across different classification thresholds

There was a difference between the reduced model with triage level only and the triage level + POC lactate model (LR test, p < 0.001), and the total fraction of new information when adding POC lactate to the triage system was 4% (1 − (0.024/0.025)). Both Nagelkerke’s pseudo R^2^ and Somers’ Dxy were lower than those of the full model, with little absolute difference between triage level and triage level + POC Lactate (Table 2). In terms of discrimination, the concordance indices (c-index) for the five models are depicted in Fig. 3. The POC Lactate as the sole predictor had the lowest performance.

## Discussion

Our findings indicate that inclusion of POC lactate in the model resulted in a marginal enhancement of the model’s prediction power in a general adult EMS population, as evidenced by a 1.5% increase in information when added to the base model and a 4% increase in information when added to the triage level alone. Use of POC lactate level as the sole predictor revealed an almost complete chance of discriminatory performance. This incremental increase in the explained variation, albeit statistically significant, suggests that the contribution of POC lactate to the overall predictive performance of the model was relatively low. These findings emphasise the importance of using POC lactate tests based on limited indications addressing more specific clinically relevant aims.

From a prehospital perspective, relevant variables in the clinical setting, together with a triage system already utilised in the prehospital setting, may have contributed to our findings. For example, patients with deviating VS or those assessed as emergent by the ambulance nurse with the aid of the triage system were identified as being at risk without the addition of POC lactate. This finding has also been reported in other studies. For instance, Wallgren et al. reported that lactate does not significantly increase model performance in predicting sepsis [18]. Moreover, in patients with an observed deterioration in the prehospital setting, despite elevated lactate levels, the biomarker was not superior in predictive ability compared with other assessment tests, such as the critical illness score [31]. In another study from Denmark involving physician-manned units, an elevated POC lactate level was associated with a higher risk of 7-day mortality [32]. However, unlike our study, which involved a more general EMS population, the Danish study reported a 7-day mortality rate of 20%, suggesting a case-mix with more severe patient conditions.

The timely identification of time-sensitive conditions is crucial for improving patient outcomes. However, this is challenging in patients with an elevated lactate level not indicative of a time-sensitive condition. This is consistent with other findings in EMS patient populations, where neither lactate levels nor urokinase plasminogen activator had significant implications for patients with non-specific complaints, even though some were later identified as having time-sensitive conditions [33]. Additionally, previous studies have shown that among patients who experienced transient loss of consciousness, those with seizures had significantly higher lactate levels compared to those with syncope or psychogenic origin [17, 34]. This can partly explain the variation among patients with self-terminated seizures with higher lactate levels later assessed as not time-sensitive or patients with time-sensitive conditions where the prehospital staff encounter the patient in an early stage in the course of illness with low levels of lactate and/or in combination with time-sensitive conditions where blood lactate is insignificant, such as stroke and transient ischaemic attack (TIA). The absence of a significant association between blood lactate levels and stroke or TIA highlights the inconsequential role of blood lactate levels in these conditions [35]. Moreover, a previous study reported that persons 65 years and older who were septic and did not survive to 28 days had a 1 mmol/L lower serum lactate level than non-survivors aged < 65 years [36]. In our study, the median age was 75 years, which may have contributed to our findings.

The present study had some limitations. First, the decision to obtain POC lactate was based on the assessment undertaken by the EMS nurse; in critically ill patients such as for example patients with a cardiac arrest, obtaining a blood test may not have been feasible, and this may have potentially biased the selection of the study population. However, such patients were likely to be transported to the emergency room with a pre-notification alert. We can´t rule out that this may have affected our findings. However, the aim was to include patients with a potential time-sensitive condition, thus excluding those with an obvious time-sensitive condition as well as those with a very low risk (triage level green without elevated respiratory rate). Second, the ICD diagnosis code stated by the discharging senior consultant was entrusted to the hospital ward. The diagnosis of sepsis was not fully represented in the ICD diagnosis, i.e. not all patients with sepsis received such an ICD code. Therefore, the use of the SOFA score in the Sepsis-3 criteria was deemed sufficient to account for these circumstances. The assessment indicative of infection was based on a workup by the ED physician. In cases in which the patient was discharged from the ED and an ICD code was not recorded, we assumed that they were cleared of any serious illness, although we lacked information on eventual re-attendance to the ED.

In terms of generalisability our results may be transferred to the rest of Sweden since EMS systems in various parts of the country are very similar and they follow in principle the same guidelines. Outside Sweden, the clinical routines are different and therefor it is not possible to speculate about the value of our findings outside Sweden.

Finally, no power calculation was performed since there was a knowledge gap regarding endpoint rate. We therefor decided to include a sample size large enough to make realistic estimations according to our statistical advisors.

## Conclusions

In a general adult EMS population in the Västra Götaland region of Sweden (EMS Gothenburg and Skaraborg), POC lactate measurement yielded limited incremental information. As an isolated predictor, the predictive efficacy of lactate level appears insufficient to discern definitive positive or negative patient outcomes. However, within distinct patient cohorts, specific subgroups such as patients with seizures, the use of POC Lactate may derive discernible informational benefits.

### Supplementary Information

Supplementary Material 1

Supplementary Material 2

## Data Availability

The data supporting the findings are not publicly available, and restrictions apply. The data may be requested upon reasonable request and with permission and approval from the Departments of Prehospital Emergency Care in Gothenburg and Skaraborg.

## Abbreviations

AMLS: Advanced Medical Life-Support
CI: Confidence interval
ED: Emergency department
EMS: Emergency medical services
ESS: Emergency signs and symptoms
ICD: International classification of diseases
IQOR: Inter-quartile odds ratio
LR: Likelihood ratio
POC: Point-of-care
RETTS: Rapid emergency triage and treatment system
RN: Registered nurse
ROC: Receiver operating characteristic
SOFA: Sequential organ failure assessment
SSX: Stat Strip Xpress
TIA: Transient ischaemic attack
VS: Vital signs
