# Supplementary material for: Added predictive value of prehospital measurement of point-of-care lactate in an adult general EMS population in Sweden: a multi-centre observational study
Source: Scand J Trauma Resusc Emerg Med. 2024 Aug 20;32:72. doi: 10.1186/s13049-024-01245-7 (PMC11337621; doi:10.1186/s13049-024-01245-7)
Supplement: Supplementary file 1 — Supplementary Material 1 [file 13049_2024_1245_MOESM1_ESM.docx]

**Additional file 1**

Calibration plots displaying the calibration performance of a logistic regression model for predicting the probability of a binary outcome (e.g., presence or absence of a disease). The plot compares the predicted probabilities (x-axis) against the observed proportions (y-axis) of the outcome within equally sized groups or bins of predicted probabilities. Ideally, points should align along the 45-degree diagonal line, indicating perfect calibration where predicted probabilities match observed proportions. Deviations from this diagonal line suggest the model's calibration performance. In the plots, the models exhibits good calibration, as most points are close to the diagonal line, indicating agreement between predicted and observed probabilities.


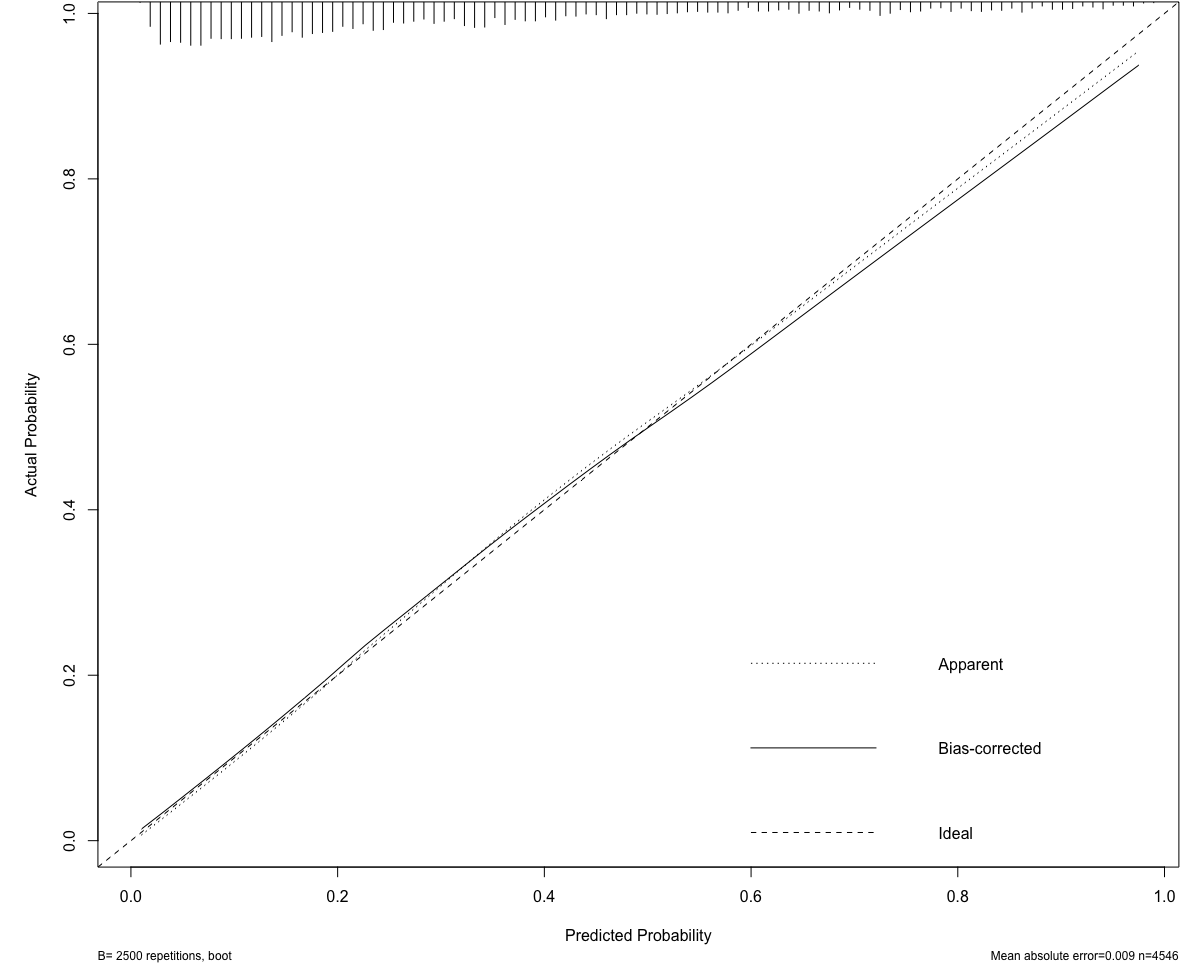


Figure 4. Calibration plot for observed and predicted probabilities of base model.


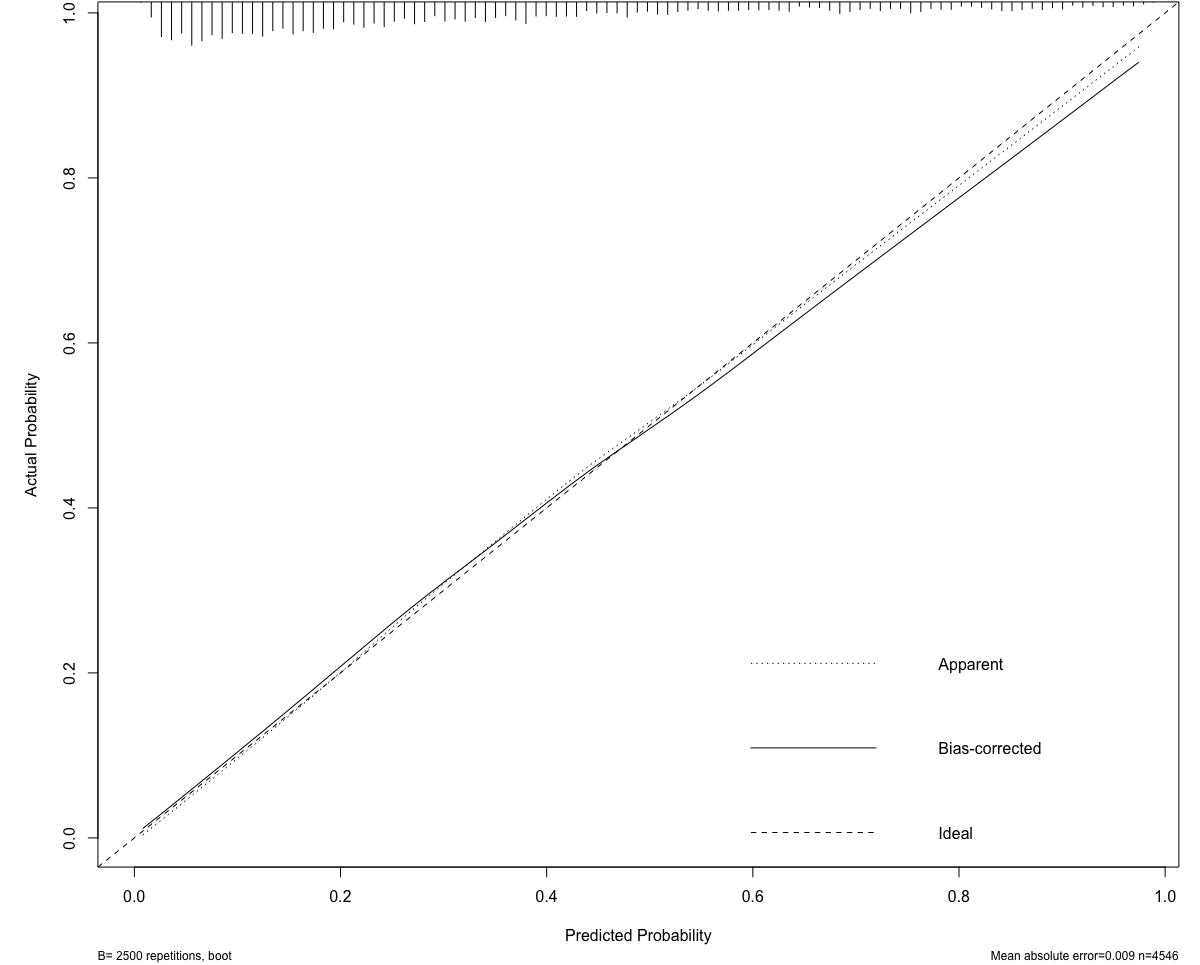


Figure 5. Calibration plot for observed and predicted probabilities of base model + POC Lactate.


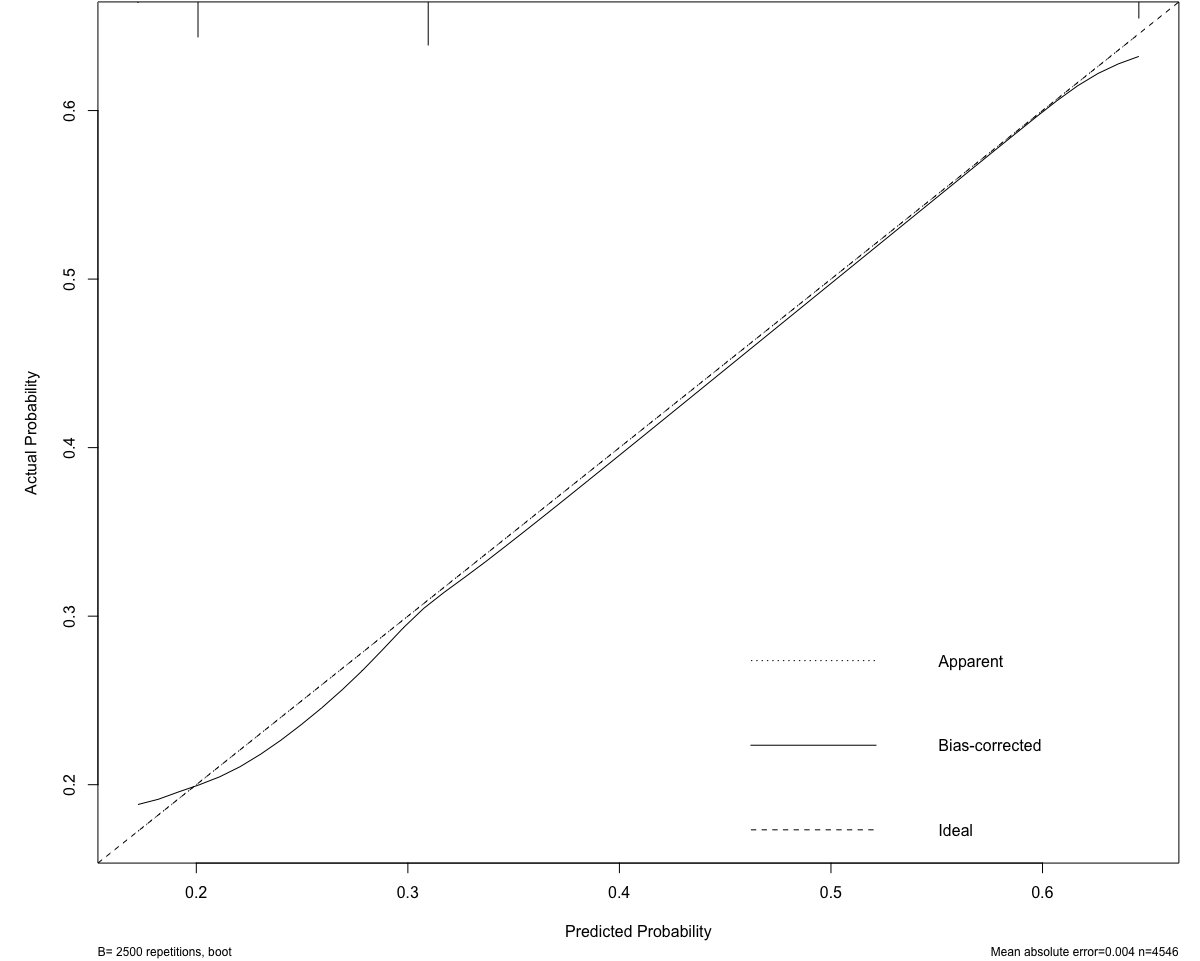


Figure 6. Calibration plot for observed and predicted probabilities of RETTS triage.


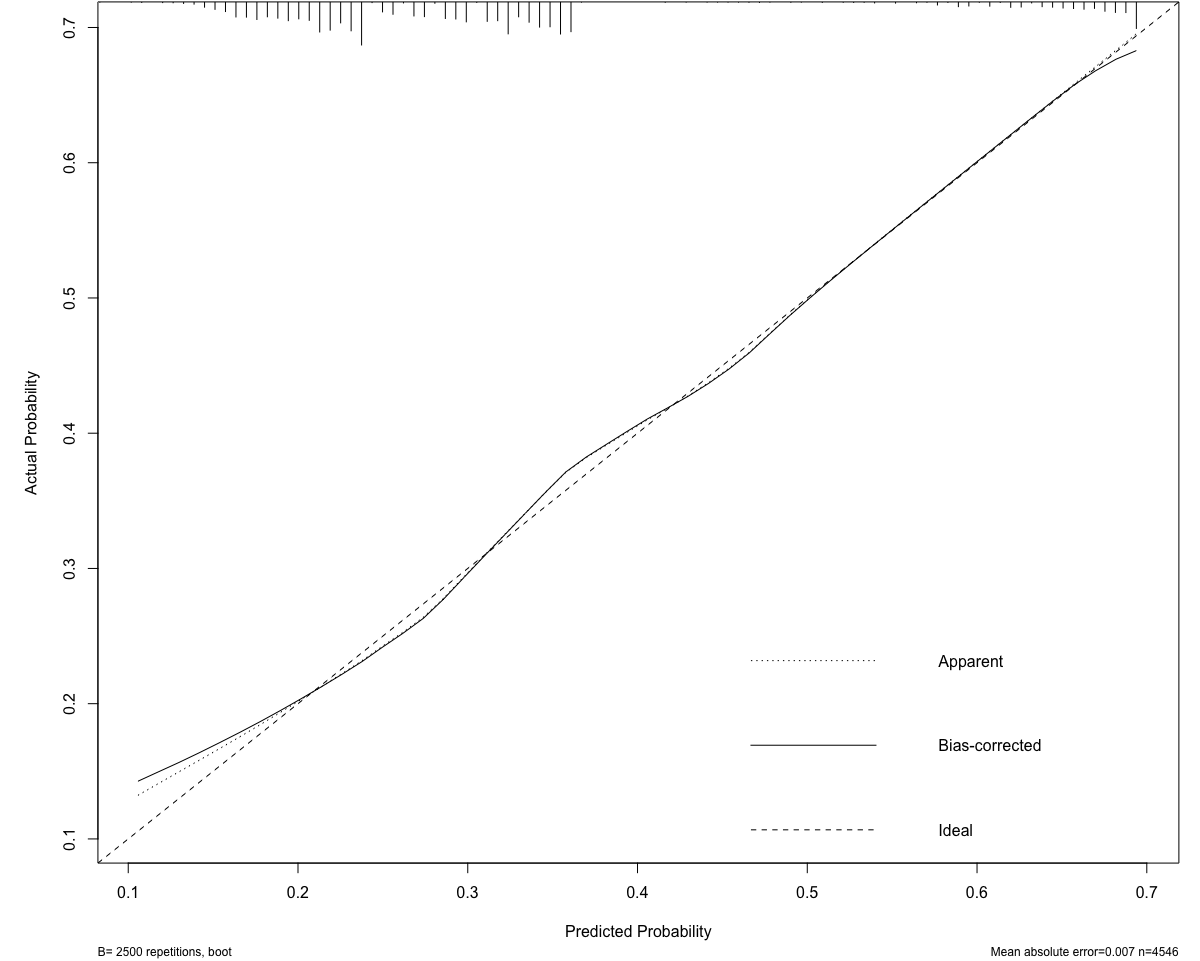


Figure 7. Calibration plot for observed and predicted probabilities of RETTS triage + POC lactate.


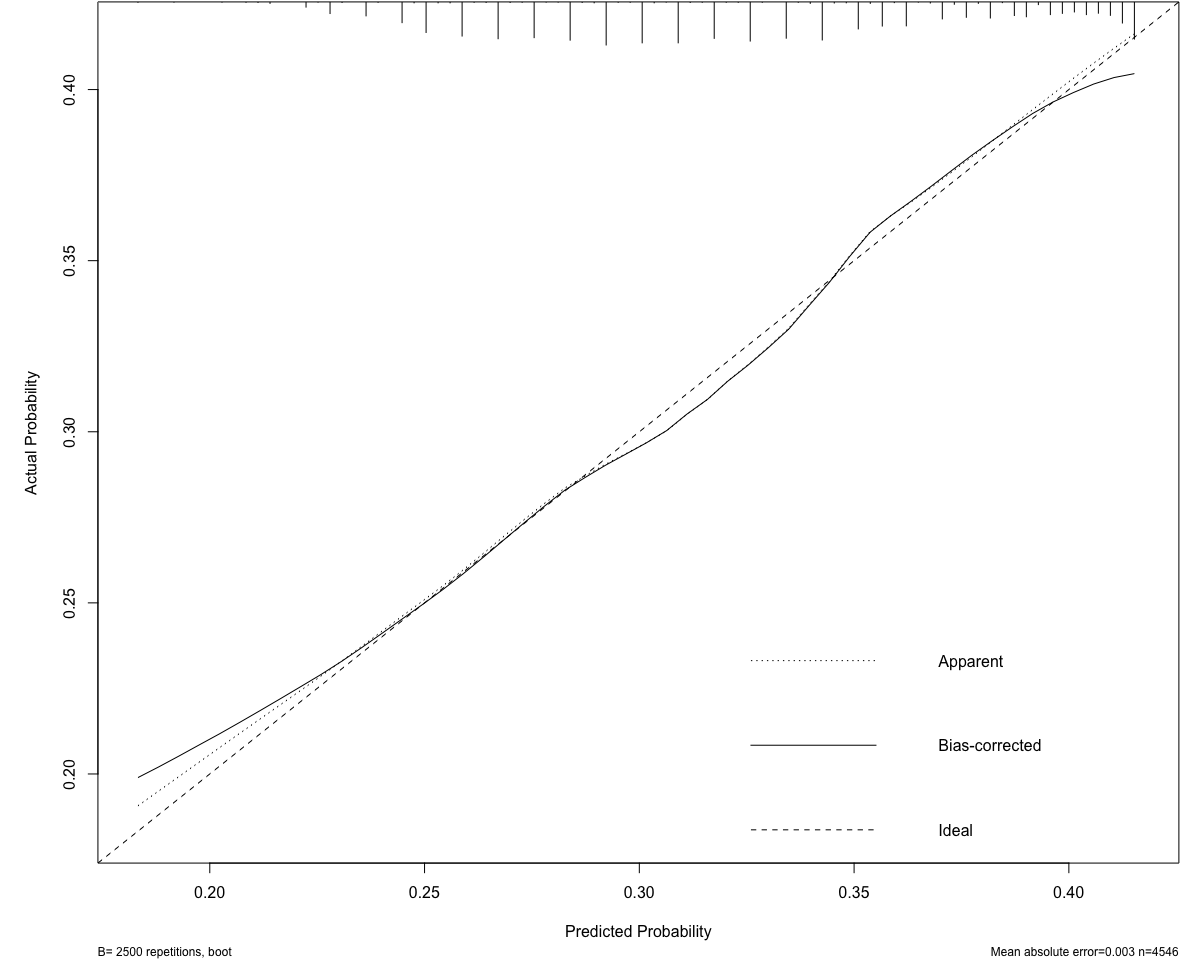


Figure 8. Calibration plot for observed and predicted probabilities of POC lactate as the only predictor.
