# Supplementary material for: Added predictive value of prehospital measurement of point-of-care lactate in an adult general EMS population in Sweden: a multi-centre observational study
Source: Scand J Trauma Resusc Emerg Med. 2024 Aug 20;32:72. doi: 10.1186/s13049-024-01245-7 (PMC11337621; doi:10.1186/s13049-024-01245-7)
Supplement: Supplementary file 2 — Supplementary Material 2 [file 13049_2024_1245_MOESM2_ESM.docx]

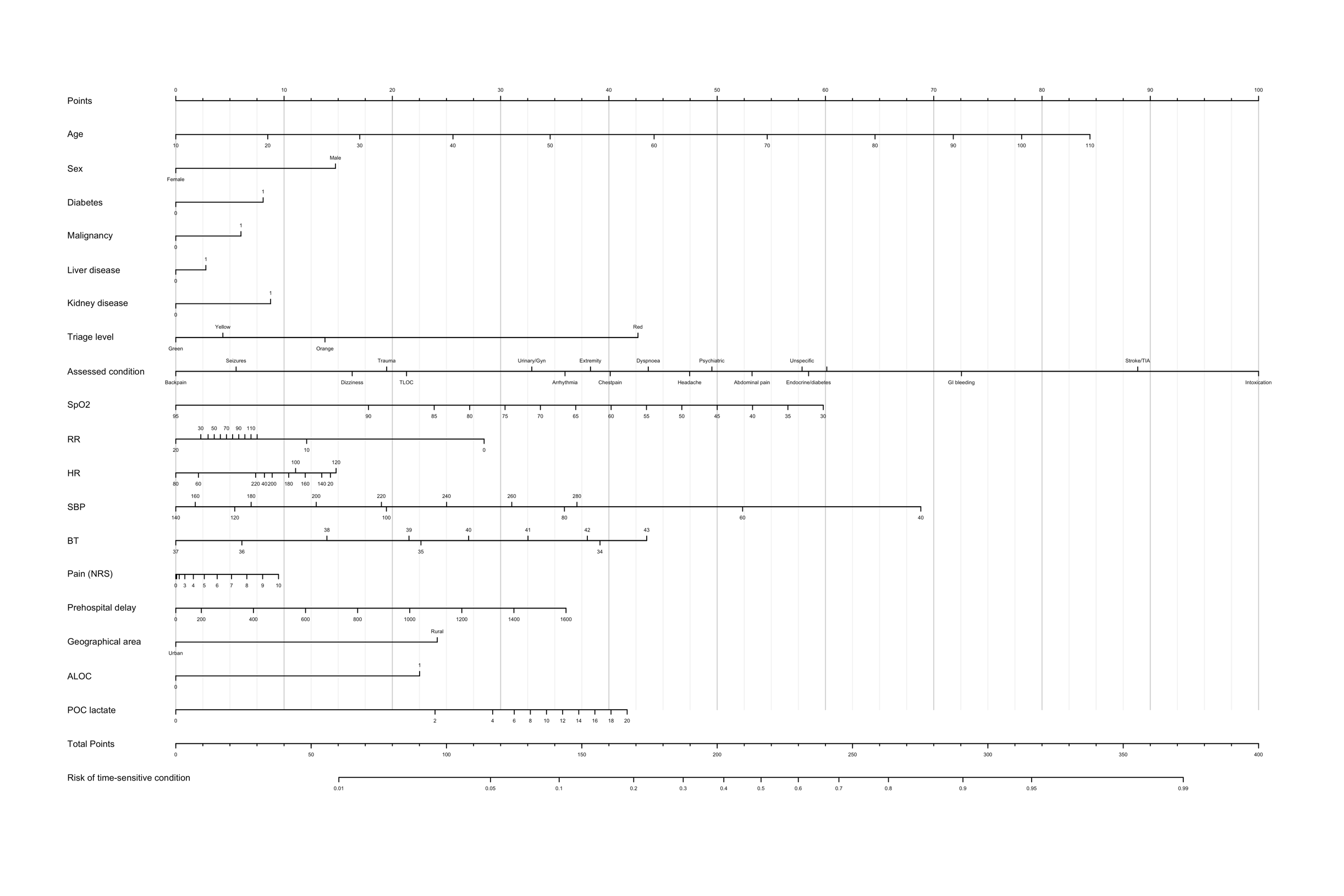
Additional file 2

Figure 9. Nomogram for the base model incorporating POC Lactate. The nomogram can be used to estimate the probability of a time-sensitive condition. This graphical tool integrates visual and numerical methods to facilitate risk assessment. To use the nomogram, locate each predictor value on and convert it to a points score by drawing a vertical line to the points scale at the top. Sum the points from all predictors to obtain a total score. Finally, use this total score on the bottom scale to determine the probability of a time-sensitive condition, providing an intuitive estimate of the patient's risk level.

Abbreviations: Age: Age in years; TIA: Transient ischemic attack; SpO2: Oxygen saturation in %; RR: Respiratory rate /min; HR: Heart rate/min; SBP: Systolic blood pressure mm/hg; BT: Body temperature in degrees Celsius; NRS: Numeric rating scale; Prehospital delay: Time in minutes from emergency call to ambulance scene arrival; ALOC: Altered level of consciousness; TLOC: Transient loss of consciousness; POC lactate: point-of-care lactate mmol/L.
